# An Upgrade on the Rabbit Model of Anthracycline-Induced Cardiomyopathy: Shorter Protocol, Reduced Mortality, and Higher Incidence of Overt Dilated Cardiomyopathy

**DOI:** 10.1155/2015/465342

**Published:** 2015-12-16

**Authors:** Jesús Talavera, Alejandro Giraldo, María Josefa Fernández-Del-Palacio, Obdulio García-Nicolás, Juan Seva, Gavin Brooks, Jose M. Moraleda

**Affiliations:** ^1^Departamento de Medicina y Cirugía Animal, Facultad de Veterinaria, Universidad de Murcia, Campus de Excelencia Internacional Regional “Campus Mare Nostrum”, 30100 Murcia, Spain; ^2^School of Biological Sciences, Institute for Cardiovascular and Metabolic Research, University of Reading, Reading RG6 6AS, UK; ^3^Departamento de Anatomía y Anatomía Comparada, Facultad de Veterinaria, Universidad de Murcia, Campus de Excelencia Internacional Regional “Campus Mare Nostrum”, 30100 Murcia, Spain; ^4^Institute of Virology and Immunology (IVI), Sensemattstrasse 293, 3147 Mittelhäusern, Switzerland; ^5^Unidad de Trasplante Hematopoyético y Terapia Celular, Departamento de Hematología, Hospital Universitario Virgen de la Arrixaca, IMIB, Universidad de Murcia, 30120 Murcia, Spain

## Abstract

Current protocols of anthracycline-induced cardiomyopathy in rabbits present with high premature mortality and nephrotoxicity, thus rendering them unsuitable for studies requiring long-term functional evaluation of myocardial function (e.g., stem cell therapy). We compared two previously described protocols to an in-house developed protocol in three groups: Group DOX2 received doxorubicin 2 mg/kg/week (8 weeks); Group DAU3 received daunorubicin 3 mg/kg/week (10 weeks); and Group DAU4 received daunorubicin 4 mg/kg/week (6 weeks). A cohort of rabbits received saline (control). Results of blood tests, cardiac troponin I, echocardiography, and histopathology were analysed. Whilst DOX2 and DAU3 rabbits showed high premature mortality (50% and 33%, resp.), DAU4 rabbits showed 7.6% premature mortality. None of DOX2 rabbits developed overt dilated cardiomyopathy; 66% of DAU3 rabbits developed overt dilated cardiomyopathy and quickly progressed to severe congestive heart failure. Interestingly, 92% of DAU4 rabbits showed overt dilated cardiomyopathy and 67% developed congestive heart failure exhibiting stable disease. DOX2 and DAU3 rabbits showed alterations of renal function, with DAU3 also exhibiting hepatic function compromise. Thus, a shortened protocol of anthracycline-induced cardiomyopathy as in DAU4 group results in high incidence of overt dilated cardiomyopathy, which insidiously progressed to congestive heart failure, associated to reduced systemic compromise and very low premature mortality.

## 1. Introduction

Anthracyclines (AC) such as doxorubicin and daunorubicin are regarded as one of the most effective chemotherapeutic groups ever developed for the treatment of malignancies, with a broad spectrum of action encompassing solid and haematologic tumours [1, 2]. Unfortunately, AC-induced cardiomyopathy (AICM) is a frequent toxic consequence of AC. It is estimated that in the USA alone there are at least 10 million cancer survivors, with a similar number in Europe [3, 4]. Thus, the recent success of chemotherapy in clinical oncology means that the population affected by AICM will likely increase substantially in the future.

Several animal models and protocols for the induction of AICM in rodents (e.g., mice and rats) and lagomorphs (e.g., rabbits) have been published over the past decades. Of note, rabbit models of cardiac disease appear to have several advantages over animal models of other species. For example, whilst being medium size animals, rabbits maintain a cellular electrophysiology and Ca^+2^ transport system, much like in the human or larger animals (e.g., dogs and pigs), which is not the case for mice and rats [5]. One of the first long-term animal models of AICM was established in rabbits by administering daunorubicin and demonstrating myocardial damage and fibrosis [6]. Subsequent studies in rabbits characterized the cumulative and delayed nature of myocardial lesions secondary to administration of doxorubicin 2 mg/kg weekly at different time points [7]. Most studies using rabbit models of AICM have focused on the evaluation of potential cardioprotective agents aimed at preventing AICM development [8–13]. However, with the current trend of increased life expectancy of cancer patients and their associated risk of long-term cardiovascular sequelae, the preclinical assessment of novel therapies (e.g., stem cell therapy) to treat this condition requires the refinement of current animal models to maximise their potential.

One of the main disadvantages of current protocols of induction of AICM in rabbits is the high mortality rate during the induction period (30–70%) [10, 14–16]. This not only increases the number of animals required but also limits their utility in the evaluation of the beneficial effects of novel therapies for AICM. Another disadvantage is that even though cardiac toxicity is readily reproducible with some protocols using daunorubicin or doxorubicin concomitant systemic toxicity (e.g., nephrotoxicity) occurs with these protocols of induction [10, 17–20]. This systemic toxicity is often responsible for the premature death of the animals even before they can develop clinically evident signs of overt dilated cardiomyopathy (DCM) and congestive heart failure (CHF). The availability of an experimental model that provides animals in a stable clinical stage of heart failure would offer a valuable tool to researchers interested in evaluating the benefits of therapies that require long-term follow-up of the animals.

Our goal has been to study the benefits of stem cell therapy in AICM, but in the process we have come across the pitfalls of current protocols of induction, thus motivating us to develop our own in-house protocol. Since cardiac toxicity is directly related to the cumulative dose administered to the subject of study [21] and rabbits are very sensitive to myocardial damage by AC [7], we hypothesised that increasing the weekly dose of daunorubicin and reducing the length of the protocol of induction could result in a rabbit model of AICM potentially exhibiting reduced nonspecific toxicities and lower mortality. Of the several experimental protocols trialled, here we report the incidence of overt DCM, CHF, premature mortality, and associated systemic toxicities when using an in-house developed shortened protocol (daunorubicin 4 mg/kg per week for up to 6 weeks) compared to other protocols reported in the literature which are frequently used for the evaluation of cardioprotective agents.

## 2. Materials and Methods

### 2.1. Animals

The experiments in the present study were performed in accordance with Directive 2010/63/EU of the European Commission and were approved by the Ethical Research Committee of the University of Murcia, Spain. A total of 37 New Zealand Rabbits (2 months old, 1.5–2.0 kg weight with 1 : 1 ratio of males/females) were randomly allocated into one of the following three groups: 6 rabbits were injected I.V. 0.9% saline weekly for up to 10 weeks and constituted the age matched controls (control group); 12 rabbits constituted DOX2 group receiving doxorubicin (Tedec-Meiji Farma, Madrid, Spain) 2 mg/kg in weekly intravenous injections for 8 weeks; 6 rabbits constituted the DAU3 group which was injected with daunorubicin (Daunoblastina, Pfizer, Madrid, Spain) 3 mg/kg in weekly for 10 weeks; and 13 rabbits constituted the DAU4 group (daunorubicin 4 mg/kg per week for 6 weeks). Due to inherent differences in the induction times for each protocol, three time points were defined for the comparisons of biochemical, haematological, and echocardiographic analyses between study groups: the first time point is the baseline time point, just before starting the first administration of anthracycline in treated groups or saline in control group; the second time point is the intermediate time point, two weeks before the end of the protocol; thus intermediate time points were at 8 weeks for control group, 6 weeks for DOX2 group, 8 weeks for DAU3 group, and 4 weeks for DAU4 group; and the third time point is the final time point, the last measurement taken upon completion of the protocol.

### 2.2. Mortality

The premature death of the animals during the period of induction of cardiomyopathy (i.e., death before completing the corresponding experimental induction protocol of weekly AC injections) was classified as follows: (1) deaths due to systemic toxicity (i.e., directly related to the administration of AC) which manifested as diarrhoea, weight loss, and emaciation and included animals that died or required euthanasia for this cause, (2) deaths secondary to CHF (see below), which were confirmed either by echocardiogram and/or by autopsy, (3) other causes such as respiratory failure during anaesthesia or animals that required euthanasia (e.g., spontaneous spinal fracture). Kaplan-Meier survival analysis was performed throughout the induction period and up to two weeks after completion of the respective induction protocol for each group.

### 2.3. Incidence of Overt DCM and CHF

**Figure 1:**
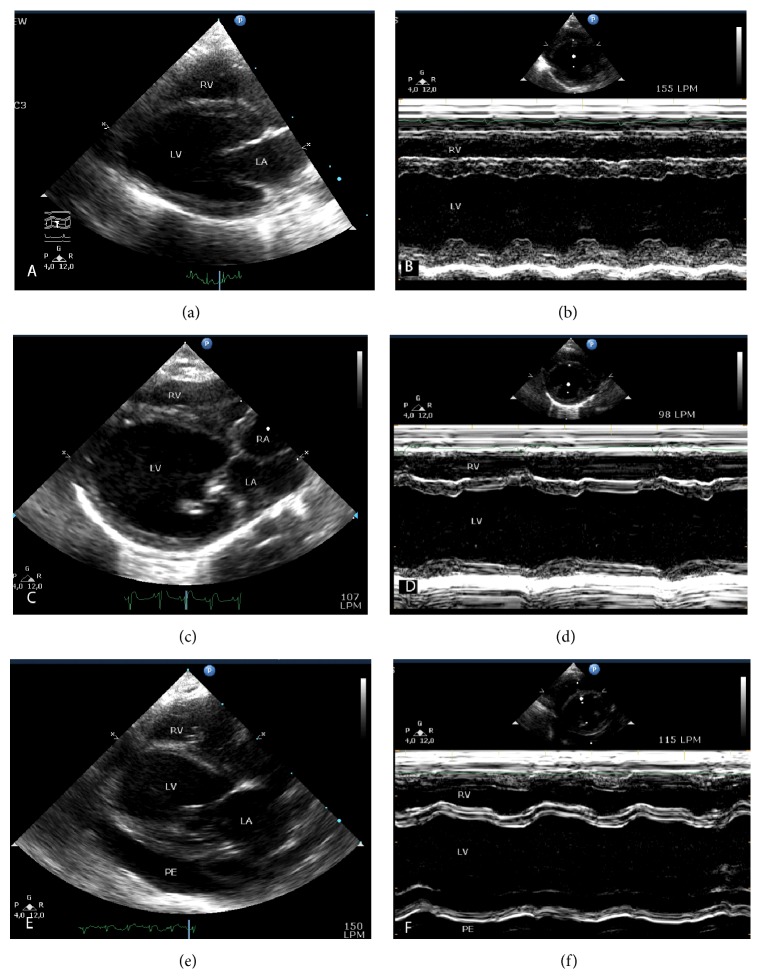
Echocardiograms in 2D and M-mode during induction of AICM. Echocardiograms in 2D parasternal long axis view (a, c, e) and M-mode of the left ventricle (LV) at the level of the papillary muscles (b, d, f). (a-b) Baseline, normal rabbit. (c-d) Rabbit with overt DCM. (e-f) Rabbit with CHF showing 4-chamber dilatation, pericardial effusion (PE), and severely reduced LV contractility. RV, right ventricle; LA, left atrium; RA, right atrium.

For the analysis of incidence of overt DCM and development of CHF in the different groups of study, these were defined as follows. Overt DCM is defined as unequivocal echocardiographic signs of cardiac remodelling and/or functional impairment objectively assessed by the presence of several of the following findings: eccentric hypertrophy of cardiac chambers, loss of the oval shape of the left ventricle, ventricular wall thinning, increased left atrial-to-aortic root ratio >  1.5 (2D-mode, short axis view), fractional shortening (FS) < 20%, left ventricular ejection fraction (LVEF) < 40%, and presence of atrioventricular valve regurgitation (assessed by colour and/or spectral Doppler) (Figures 1(c) and 1(d)). On the other hand, CHF was defined as echocardiographic and/or postmortem evidence of pleural, pericardial, and peritoneal effusions and/or pulmonary oedema in conjunction with echocardiographic signs of overt DCM and/or severe cardiomegaly confirmed by postmortem study (Figures 1(e) and 1(f)).

### 2.4. Administration of Drugs and Blood Sampling

Animals were immobilised in acrylic restrainers to reduce stress and facilitate the administration of drugs. AC were diluted according to the weight of the animal in 8 mL of 0.9% sterile saline for I.V. injections in AC treated groups, whilst in control group 8 mL of 0.9% sterile saline without AC were injected at each time point. After hair clipping of the ear and careful asepsis, a 24 G catheter was advanced into one of the marginal auricular veins and fixed with micropore to the skin. A winged needle (21 G) with prolongator was then inserted into the catheter and AC were administered at a rate of 0.5 mL/min. Blood samples from jugular vein were collected before first dose of AC and then every 2 weeks until the final administration at 8 weeks (DOX2 group), 10 weeks (DAU3 group), or 6 weeks (DAU4 group).

### 2.5. Biochemical and Haematological Study

Haematological parameters in plasma samples were analysed using the Advia 120 Hematology System (Siemens, Erlangen, Germany). Biochemical parameters were evaluated in plasma using the analyser AU2700 (Olympus Corporation, Tokyo, Japan).

### 2.6. Echocardiographic Study

A transthoracic echocardiographic examination using a HD7 XE System, equipped with a 4–12 MHz transducer (Philips, Andover, Massachusetts, USA) under light anaesthesia (ketamine (Imalgene, Merial, Villeurbanne, France) 10 mg/kg, combined with dexmedetomidine (Domtor, Esteve, Madrid, Spain) 200 *μ*g/kg), was performed longitudinally at baseline and every two weeks until the end of the study. The procedure was performed by or under direct supervision of a European board-certified veterinary cardiologist (MJFP), in a blinded fashion, according to the recommendations of the Echocardiography Committee of the American College of Veterinary Internal Medicine and the American Society of Echocardiography [22, 23]. Simultaneous 1-lead electrocardiographic tracings were recorded during the study. Total circumferential shortening area (CSA) was obtained using the following formula: CSA = CSAd − CSAs/CSAd × 100. Fractional shortening (FS (%)) was calculated according to the following formula: FS = (LVDd − LVDs)/(LVDd × 100). Left ventricular systolic and diastolic volumes (LVVd and LVVs) were calculated using the Teichholz formula (7 × (LVD)^3^)/(2.4 + LVD) and LVEF (%) was calculated according to the following formula: LVEF = (LVVd − LVVs)/(LVVd × 100).

### 2.7. Cardiac Troponin I Evaluation

Cardiac troponin I (cTnI) levels were determined in plasma samples obtained from the jugular vein using the Immulite Kit (Siemens, Germany) according to the manufacturer's instructions with a detection limit <0.049 ng/mL.

### 2.8. Histopathological Study

Heart tissue blocks were sectioned to cover 5 different anatomical regions in ascending order: the apex, free wall bellow the papillary muscles, at the level of the papillary muscles, end of papillary muscles and beginning of* chordae tendineae*, and at the atrial level [7]. Tissue was fixed for 24 h in 10% formalin, dehydrated with increasing ethanol concentrations, which was then substituted for xylene, and finally embedded in paraffin using the Leica TP1050 cyclic tissue processor (Leica Biosystems GmbH, Germany) and the Tissue-Tek thermal console (Sakura, USA). Sections of 5 *μ*m were then obtained with a microtome RM2155 (Leica Biosystems GmbH, Germany) and stained with haematoxylin-eosin and Masson's trichrome. Two pathologists blinded to study group performed the histopathological evaluations. Myocardial lesions were assigned a score from 1–4 by lesion grade, and the extension of myocardial lesions was classified 1–4 as previously described [7]. A similar lesion grade scoring (1–4) and classification of extension of lesions (1–4) system was followed for kidney and liver tissue.

### 2.9. Statistical Analysis

Statistical analysis was performed using SPSS Statistics version 19 for Windows and GraphPad Prism 6. Data are expressed as means ± SEM. One-way ANOVA with Tukey's post hoc test or paired *t* test/Wilcoxon signed-rank test or two-way ANOVA to assess the global effect of sex and treatment, where appropriate, was performed. Survival curves were plotted using Kaplan-Meier analysis (Log-Rank with Mantel-Cox test). Values of *p* ≤ 0.05 were considered statistically significant.

## 3. Results

### 3.1. Mortality

**Figure 2:**
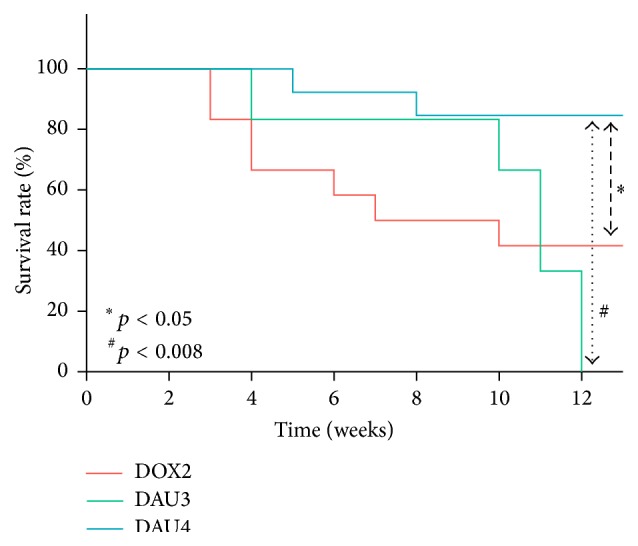
Kaplan-Meier survival curves.

Survival analysis curves are shown in Figure 2. In the DOX2 group, 6 out of 12 animals (50%) died prematurely (premature mortality defined as death of the animal during the course of weekly I.V. administrations on each group) during weeks 3–7 of the induction period (up to 8 weeks), all secondary to systemic toxicity. In the DAU3 group, 33.3% died prematurely during the induction period (up to 10 weeks); one of these deaths occurred secondary to systemic toxicity at 4 weeks and the other occurred secondary to CHF at week 9. In the DAU4 group, only one animal required euthanasia at week 5 due to spontaneous spinal fracture resulting in a premature mortality of 7.6% during the induction period (up to 6 weeks). No deaths occurred in the control group. Gender of the animal did not influence mortality in all AC treated groups since this did not differ substantially from that of whole cohort within each group. Thus, of the six deaths in group DOX2 during the induction period, three corresponded to males and three to females. Similarly, one death occurred in males and one in females in the DAU3 group during the induction period, whilst for the DAU4 group the premature death occurred in a male rabbit. Cumulative mortality up to two weeks after completion of the induction period continued to increase in all groups (Figure 2). However, whilst one additional rabbit died in the DOX2 group from 8 to 10 weeks, secondary to systemic toxicity, and 4 more deaths occurred in the DAU3 group from 10 to 12 weeks, 3 of these secondary to CHF, resulting in a cumulative mortality of 58.3% and 100%, respectively, only one more death secondary to CHF occurred in the DAU4 group from 6–8 weeks, resulting in a cumulative mortality of 15.3%.

### 3.2. Incidence of DCM and CHF

None of the animals from the DOX2 group developed overt DCM or CHF, which on the contrary were greatly affected by general toxicity. In the DAU3 group, 4 out of 6 animals (66%) developed overt DCM and CHF, of which two were males and two were females. In the DAU4 group, 12 out of 13 animals presented with overt DCM (92%), whilst CHF was confirmed in 8 animals (61%). In DAU4 group, the incidence of overt DCM and CHF according to sex of the animal revealed that 6 out of 7 males and 6 out of 6 females presented with overt DCM, whilst HF was present in 4 out of 7 males and 4 out of 6 females.

### 3.3. Biochemical Study

Selected biochemical parameters are shown in Table 1. No differences were observed between the different groups at baseline. Triglycerides and cholesterol plus triglycerides were significantly increased at the intermediate time point in the DOX2 and DAU3 groups, respectively, compared to basal values and control group, whilst in the DAU4 group no significant differences were observed. Also, there were slight increases in other biochemical parameters such as creatinine for DOX2 and DAU3 groups at the intermediate time point; however, these remained within the normal range for the species [24]. Groups DOX2 and DAU3 developed marked changes in several biochemical parameters at the final time point of the study. Thus, rabbits in the DOX2 and DAU3 groups exhibited a significant increase in creatinine and marked combined hyperlipidaemia associated with a concomitant reduction of total proteins. In addition, the DAU3 group also showed significant increases in blood urea nitrogen (BUN), aspartate aminotransferases (AST), and alanine aminotransferases (ALT). Apart from mild combined hyperlipidaemia, no significant changes were observed in biochemical parameters in the DAU4 group at the final time point (Table 1). Supplemental Table  1 in Supplementary Material available online at http://dx.doi.org/10.1155/2015/465342 shows the subanalysis of selected biochemical parameters in male and female animals from all groups at final time point. Consistent with the general analysis, both male and female rabbits in the DOX2 and DAU3 groups had comparable increases in creatinine values, marked combined hyperlipidaemia, and reduction of total proteins in blood relative to control group, suggesting that none of these alterations had an underlying causality associated with sex of the animal. However, even though these changes were marked enough compared to control group, they were only significant in DOX2 group, provided the low *n* of animals in DAU3 group at this time point (type II error). Similarly, and in line with the findings of the general analysis, DAU3 group also presented marked alterations in BUN, AST, and ALT values in both males and females, although low *n* at this time point in DAU3 group leads to type II error (Supplemental Table  1). Male and female rabbits from the DAU4 group only exhibited mild hyperlipidaemia.

### 3.4. Haematological Study

In all AC treated groups, a significant reduction in red blood cells (RBC), haemoglobin, and haematocrit and increased red cell distribution width (RDW) were observed at the intermediate time point relative to basal time point and control group. However, whilst in groups DOX2 and DAU4 these parameters returned to normal levels at the end of the induction period, these remained abnormal or even worsened in the DAU3 group at the end of the induction period (Table 2). Consistent with increased haematological toxicity with the DAU3 protocol, white blood cell (WBC) count also was significantly affected at the intermediate time point compared to baseline values and those of control group, although these values returned to nearly normal at the end of the induction period (Table 2).

### 3.5. Echocardiographic Study

**Figure 3:**
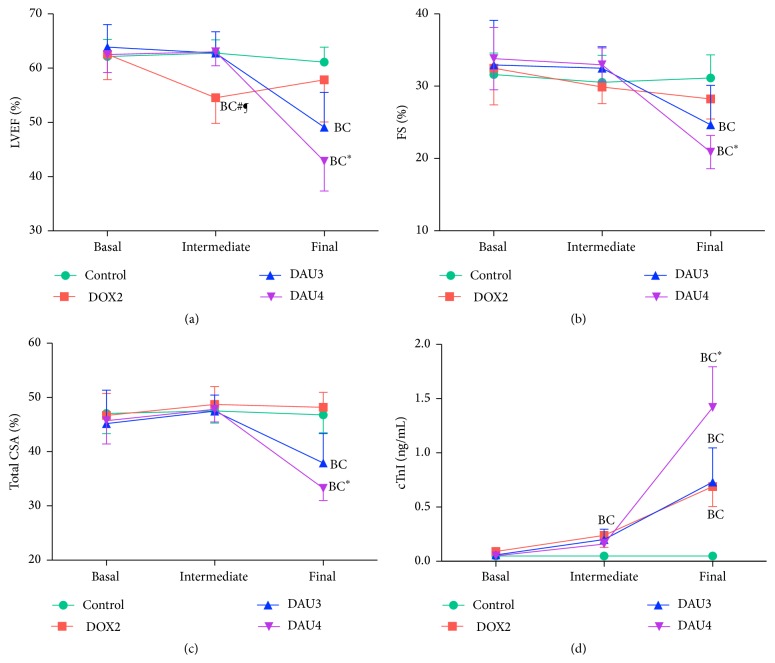
Changes in echocardiographic parameters and cTnI values during induction of AICM. (a) Left ventricular ejection fraction (LVEF). (b) Fractional shortening (FS). (c) Total circumferential shortening area (CSA). (d) Cardiac troponin I (cTnI) levels. Data expressed as mean ± SEM. Statistical significance is at *p* < 0.05; (B), compared to basal; (C), compared to control; (*∗*), compared to DOX2; (#), compared to DAU3; (¶), compared to DAU4.

All groups of the study had comparable LVEF, FS, and total CSA at basal time point (Figures 3(a)–3(c)). A repeated measures test within the control group demonstrated no significant differences on these parameters of ventricular function at the different time points of the study. In the DOX2 group, a decrease in LVEF and FS was observed at the intermediate time point (6 weeks) and final time point (8 weeks), but this was only statistically significant for LVEF at the intermediate time point (Figures 3(a) and 3(b)). Whilst at the intermediate time point LVEF, FS, and total CSA remained unchanged in DAU3 and DAU4 groups compared to basal time point and control group, all these parameters of ventricular function were markedly and significantly reduced in rabbits from these groups at the final time point (Figures 3(a)–3(c)). These changes were also significantly different in DAU4 group when compared to DOX2 at this time point (Figures 3(a)–3(c)). Subanalysis of ventricular function in all groups at the final time point indicated that AC-induced myocardial damage affected males and females in DAU3 and DAU4 groups to a similar extent. Thus, marked alterations in LVEF, FS, and total CSA in males and females were observed at final time point (Supplemental Figures  1(A)–1(C)). However, whilst these changes were significant in DAU4 group, low *n* at final time point in DAU3 group resulted in type II error. On the contrary, ventricular function remained equally unaffected in males and females from DOX2 group (Supplemental Figures  1(A)–1(C)).

### 3.6. Cardiac Troponin I Evaluation

At the basal time point, no differences in cTnI levels were observed among all groups. Significant elevations were observed at intermediate time points in all AC treated groups relative to basal time point and control group (Figure 3(d)). The levels of cTnI further increased at the final time point in all groups, although the most marked increase was observed in the DAU4 group to the extent of being statistically significant at this time point compared to values for the DOX2 group (Figure 3(d)). The cTnI levels in males and females were also analysed at final time point in all groups (Supplemental Figure  1(D)). In line with the findings for the general population, marked elevations of cTnI were observed in both males and females from all groups, whilst males and females from DAU4 group exhibited the highest levels of cTnI elevation relative to control (Supplemental Figure  1(D)).

### 3.7. Histopathological Examination

**Figure 4:**
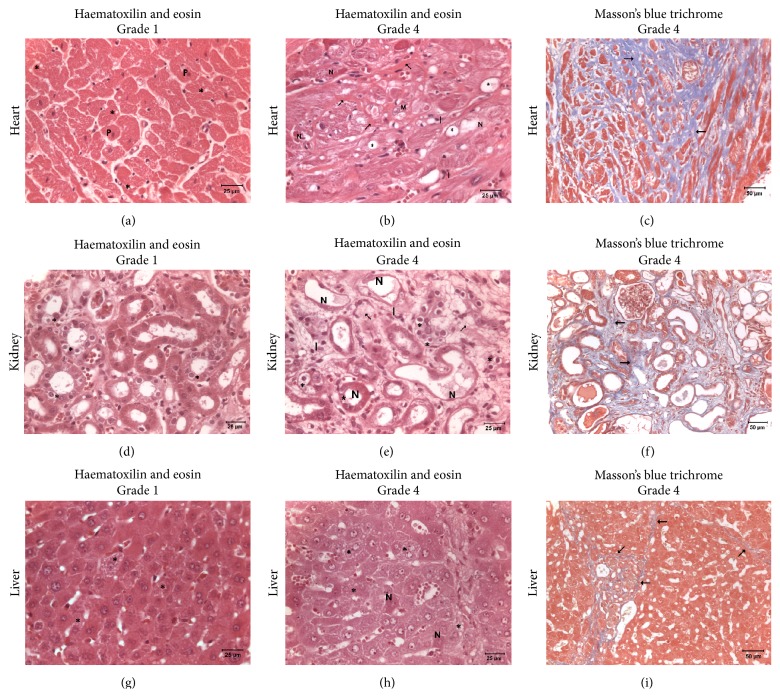
Myocardial, renal, and hepatic lesions induced by anthracyclines. (a–c) Representative photomicrographs of myocardial damage. (a) DOX2 group, grade 1: primary damage of the left ventricular myocardium. Highly intense eosinophilia of the cytoplasm, pyknotic nuclei [P], and diffuse vacuolar degenerations in cardiomyocytes [*∗*] are present. (b) DAU3 group, grade 4: conspicuous disperse toxic myocardial damage. Extensively vacuolated cardiomyocytes [*∗*] with intense eosinophilic cytoplasm are present. Necrotic cardiomyocytes [N] phagocytized by macrophages [M] were gradually replaced by proliferated connective tissue (e.g., thick wavy collagen fibers [arrows]). Mononuclear infiltrate [I] is present within the bundles of these fibers. (c) DAU3 group, grade 4: extensive fibrosis [arrows] in areas with myocytolysis and necrosis of cardiomyocytes. (d–f) Representative photomicrographs of renal damage. (d) DOX2 group, grade 1: initial renal tubular necrosis. Lesions of nephrosis with tubular degeneration [*∗*] are present. (e) DAU3 group, grade 4: focal severe nephropathy. Lesions of nephrosis with tubular degeneration [*∗*] and extensive necrosis [N] of tubular cells are frequent. Fibrosis [arrow] and mononuclear infiltrate [I] are present in these areas. (f) DAU3 group, grade 4: extensive fibrosis [arrows] in areas of toxic nephropathy with degenerations and tubular necrosis. (g–i) Representative photomicrographs of hepatic damage. (g) DOX2 group, grade 1: initial damage of the liver. Some hepatocytes show diffuse vacuoles inside cytoplasm [*∗*]. (h) DAU3 group, grade 4: hepatocytes revealed an extensive toxic damage. Necrotic hepatocytes are present [N] and abundant hepatocytes show numerous and extensive vacuoles inside cytoplasm [*∗*]. (i) DAU3 group, grade 4: diffuse fibrosis [arrows] in areas with necrotic hepatocytes. (a-b), (d-e), and (f-g): haematoxylin and eosin; (c), (f), and (i): Masson's blue trichrome.

Figure 4 shows representative photomicrographs of grades 1 and 4, within the spectrum of AC induced lesions in the myocardium, kidney, and liver, according to the grading scale used to determine the score of lesions per organ [7]. The myocardial lesions from all groups showed similar scores in grading scale as shown in Table 3. Thus, the myocardium of most animals presented predominantly with moderate myocytolysis associated with atrophy and degeneration of myofibrils and replacement fibrosis (Figures 4(b) and 4(c)). However, the extent of myocardial lesions was different for all groups. Whilst DOX2 group animals presented mostly circumscribed lesions in isolated myofibrils, myocardial lesions in animals from the DAU3 group affected focal groups of myofibrils at 1 level, and animals of the DAU4 group exhibited lesions involving more extensive (diffuse) groups of myofibrils at 2 or more levels (Table 3). Nonpurulent myocarditis associated with the presence of mononuclear cell infiltration was also a frequent observation in animals from all groups. Kidney and liver lesions from all groups showed similar scores, as well as similar extension of lesions (see Table 4).

## 4. Discussion

An animal model of AICM suitable to test novel therapies aimed at ameliorating this condition not only should reproduce the cardiomyopathic effect of AC in scheduled intravenous injections, thus simulating clinical scenarios [25], but also should have low mortality at the end of the induction period, thereby allowing sufficient time to complete experiments to evaluate their efficacy in recovering myocardial function and animal well-being. It also is desirable that nonspecific toxicities, such as nephrotoxicity, which do not occur in humans treated with AC [18], are reduced or absent from the model to avoid the potential confounding effect of comorbidities in the outcome of the experiments. Such an animal model is currently unavailable. The present study presents an experimental protocol for AICM that generates a high percentage of animals with overt DCM and CHF with mild manifestations of systemic toxicity and very low mortality both premature (7.6%) and within two weeks of completing the induction protocol (15.3%). Furthermore, the occurrence of CHF manifests insidiously whilst cardiogenic death did not occur abruptly at the end of the induction period, thus allowing time for functional evaluation of the animal over an extended period of time. These qualities result in an improvement over previously published protocols of AICM in rabbits particularly in the setting of evaluation of novel therapies for AICM (e.g., stem cell therapy). This refined model is in line with the principles of the 3Rs and the guidelines of the National Centre for the Replacement, Refinement and Reduction of Animals in Research. Thus, lower premature mortality translates into lower number of animals required to complete a study (reduction), and reduced nonspecific toxicities translate into reduced unnecessary suffering and improved well-being of the rabbits throughout the study (refinement).

One of the salient findings of our study is that, in contrast to the high attrition rate associated with the protocols of induction used in DOX2 and DAU3 groups (Figure 2), the DAU4 group exhibited very low premature mortality (7.6%) during the induction period (with premature mortality defined as death of the animal during the course of weekly I.V. administrations on each group). DAU4 group also exhibited a significantly higher survival rate within two weeks after completion of the induction protocol (Figure 2). Mortality associated with the induction of heart failure secondary to AICM in animal models using rabbits is rarely reported. Among the studies that do report mortality using protocols of induction similar to those employed in our DOX2 and DAU3 groups, the premature mortality is consistent with our findings (30–50%) [10, 14, 19]. The significantly lower mortality in DAU4 group compared to that of DAU3 could be in part attributable to the fact that whilst higher weekly doses were administered in DAU4 (3 mg/kg/week in DAU3 compared to 4 mg/kg/week in DAU4), these were administered over a shorter period of time (10 weeks in DAU3 versus 6 weeks in DAU4). This likely minimises the period of AC exposure, thus reducing the overall total cumulative dose at the end of the induction period (30 mg/kg in DAU3 group compared to 24 mg/kg in DAU4). This resulted in comparable myocardial damage between DAU4 group and DAU3 group, whilst it reduced extracardiac toxicity/compromise in DAU4 (see also relevant discussion below). Other protocols experimentally trialled by us (e.g., 6 mg/kg/week for 4-5 weeks) resulted in mortalities above 50%, primarily due to toxicity, and were therefore stopped for ethical reasons and deemed inadequate.

Another important finding of our study is that the induction protocol used in the DAU4 group is less prone to induce nonspecific toxicity such as nephrotoxicity and hepatotoxicity compared to the induction protocols used in the DOX2 and DAU3 groups. Thus, whilst the DAU4 group did not exhibit changes in renal or hepatic function, animals from the DOX2 and DAU3 groups presented changes in renal function suggestive of nephrotic syndrome. Similarly, the DAU3 group showed increased levels of AST and ALT, suggesting compromised liver function in this group. Taking into account these findings, it is likely that the high mortality rate observed in the DOX2 and DAU3 groups is, at least in part, explained by the increased incidence of nonspecific toxic effects also observed in these groups (see also relevant discussion below). The sensitivity to the nephrotoxic effects of AC in rodents, which is not observed in humans [18], has been extensively used as a model of nephrotic syndrome which replicates most of the pathological features of focal and segmental glomerulosclerosis seen in humans [26, 27]. The nephrotoxicity observed in the DOX2 and DAU3 groups is consistent with previous reports [10, 19, 28, 29]. Hepatotoxicity induced by doxorubicin in rabbits has been described previously in one report [20]; however, we did not find alterations in biochemical markers of liver function in the DOX2 group. Interestingly, previous studies that used a protocol of induction as in the DAU3 group in the present study did not report alterations in liver function tests [10, 19]. The reasons for these differences are unknown. Nevertheless, the changes in AST and ALT at the final time point in the DAU3 group suggest marked hepatocellular damage. Whilst this could be a consequence of direct AC-induced hepatotoxicity, other mechanisms could be partially responsible as well (see also relevant discussion below). Given the shorter period of exposure in the DAU4 group and the absence of nonspecific toxicities/compromise such as nephrotoxicity and hepatotoxicity as suggested by elevations in biomarkers of kidney and liver damage, it is reasonable to conclude that these toxicities are in part related to the length of exposure to AC, as opposed to cardiotoxicity, which is directly related to the cumulative dose received, although other potential explanations are also possible (see also relevant discussion below) [21].

In our study, haematological toxicity mostly affected red blood cells and was common to all groups of this study even though for most altered parameters this was transient since this was observed at the intermediate time point and recovered to normal or near normal levels at the final time point in the DOX2 and DAU4 groups, whilst it remained altered in the DAU3 group (Table 2). Haematological toxicity consistent with myelosuppression (i.e., reduced number of RBC, WBC, and platelets) has been reported previously in patients treated with AC [30, 31] and appears to be a common feature of many antineoplastic drugs [32]. We did not observe alterations in platelet count in any of the groups studied and only transient leukopenia in the DAU3 group, which is consistent with previous studies using protocols of induction similar to those used in the DAU3 group [10, 19]. The finding that this is also observed in the DAU4 group suggests that this toxicity is independent of the scheduled dosing of AC. Indeed, given the high activity of AC in haematological malignancies, this toxicity could be related to their therapeutic benefits [1, 2].

Evaluation of left ventricular function by echocardiography is a reliable noninvasive method for diagnosis of AICM, which has good correlation with invasive catheter-based functional analysis in rabbit models of AICM [10]. It is interesting to note that in this study the echocardiographic studies were planned and supervised by European board-certified veterinary cardiologists with extensive clinical experience, which adds value to the results and ensures the rigorousness of the studies. Monitoring of LVEF and FS demonstrated progressive reduction in these parameters only in the DAU3 and DAU4 groups. On the other hand, with the exception of a transient decline in LVEF at the intermediate time point (6 weeks) in animals of the DOX2 group, these parameters remained almost unchanged throughout the induction period, and none of the animals from this group developed overt DCM or CHF. This is in contrast to a previous study by Gava et al. who used an induction protocol as in the DOX2 group in our study, which found a progressive decline in both LVEF and FS from as early as 6 weeks [15]. Similar to our findings, another study, using even higher doses of doxorubicin (doxorubicin 3 mg/kg per week for 10 weeks), also failed to demonstrate significant changes in left ventricular function [19]. The reasons for these discrepancies are unknown at present, however, since our histopathological studies indicate that this group exhibited the lowest score of extension of myocardial damage (see also relevant discussion below); the cause of death in this group was exclusively due to systemic toxicity, and mortality in the study of Gava et al. was 70% (causes and timing of these deaths were not reported) as opposed to 50% in our study [15]; we suggest that this protocol of induction may not be suitable for exploring the potential benefits of novel therapies for HF secondary to AICM.

The incidence of overt DCM for the DAU3 and DAU4 groups was 66% and 92%, respectively, which ultimately translated to an incidence of CHF of 66% (group DAU3) and 61% (group DAU4). Of note, whilst the development of overt DCM and CHF in the DAU3 group was simultaneous in all cases, this was usually full blown severe CHF and very quickly (within a couple of days) resulted in the death of the animal, in contrast to a more insidious presentation in the DAU4 group, in which incidence of overt DCM was very high followed in most cases within days by CHF, which persisted for several weeks before death ensued. Other parameters of myocardial damage were also more exacerbated in DAU4 group. Thus, whilst an elevation of cTnI, a marker of myocardial cellular damage, was observed in all groups, much higher levels were detected in the DAU4 group at the final time point (Figure 3(d)). Of note, none of the differences observed in LVEF, FS, total CSA, or cTnI between DAU3 and DAU4 groups were statistically significant, despite a clear trend (Figure 3). Elevation of cTnI has been documented previously in rabbits treated with an induction protocol as in the DAU3 group in our study [33].

Histopathological examination revealed that whilst animals of all groups have a similar grade of myocardial lesions, these were more conspicuous in the DAU4 group, since the score of extension of lesions observed in this group was significantly higher than that in the other groups (Table 3). The histopathological findings of the heart, kidney, and liver in the DOX2 and DAU3 groups are consistent with previous reports [10, 15, 19]. Of note, despite marked elevations in surrogate biomarkers of renal injury in DOX2 and DAU3 groups and of liver injury in DAU3 group compared to DAU4 group and control group at final time point, the scores of kidney and liver lesions in grade and extension of damage amongst the AC treated groups were similar upon histopathological examination (Table 4). Whilst nephrotoxicity and hepatotoxicity have been described in rabbits treated with AC, these may not completely explain this lack of correlation between blood biomarkers and histopathology findings and it is worth elucubrating about other potential explanations. The rabbits from DOX2 group were greatly affected by general toxicity and exhibited the highest mortality (50%). Rabbits from this cohort were often observed to be asthenic, subsequently developed anorexia, and near the end of the protocol (or end of their life) appeared cachectic, which likely resulted from an increased catabolic state, leading to emaciation, a condition often associated with multisystem organ failure, including kidney damage. These observations could explain, at least in part, the elevated creatinine observed in this group. On the other hand, 66% of rabbits from DAU3 group developed overt DCM which was quickly followed by progression to a severe form of CHF, which resembled acute severe decompensated CHF, with death ensuing within days. Hepatic (retrograde) congestion could in part explain the increased elevations of ALT and AST. Hypoperfusion secondary to poor pump performance and neuroendocrine activation (which is more marked in some forms of CHF such as acute decompensated CHF) and associated peripheral and renal-splanchnic vasoconstriction could contribute to increased renal damage (even though renal damage secondary to CHF (cardiorenal syndrome) is multifactorial) [34] and thus explain to some extent elevated biomarkers of kidney injury (e.g., creatinine and BUN) in the setting of AC treatment in this group.

Some long-term association studies indicate that among childhood cancer survivors treated with AC girls appear to have increased risk of developing long-term AICM [35, 36]. Interestingly, amongst adults, men appear to be more sensitive than women to AICM [37]. Gender differences also appear to play a role in rodent models of AICM and AC nephropathy, with males and ovariectomized females being more affected than females [38–40]. Although evidence is still inconclusive, with recent metaregression analyses and association studies indicating that female sex is not a risk factor for AICM [41, 42], most of the current available evidence points to the conclusion that oestrogen could have a protective role in AICM. We did not find any clear difference attributable to gender in terms of mortality, incidence of overt DCM, or congestive heart failure, as suggested by a similar percentage of males and females from DAU3 and DAU4 groups being affected in the present study. Similarly, at the final time point, we did not find any significant differences in biomarkers of cardiac (i.e., LVEF, FS, total CSA, and cTnI levels), renal (e.g., creatinine and BUN), or hepatic (i.e., AST and ALT) function amongst males and females on each of the AC treated groups (Supplemental Table  1 and Supplemental Figure  1). Thus, in contrast to experimental studies in rodents, taken together, our data do not support a link between gender differences and AC toxic sensitivity in rabbits. Of note, beyond a clear AC mediated toxicity, our study was not powered to assess sex differences to AC sensitivity in the DAU3 group, thus limiting our conclusions in this respect.

Taken together, our results suggest that during the induction of AICM the overall extracardiac physiology of rabbits from DOX2 and DAU3 groups was more severely compromised compared to that of the rabbits from DAU4 group, whilst DAU4 induced comparable myocardial damage to that of DAU3 group (Figure 3). We believe that the refined induction protocol used in the DAU4 group could be better suited for evaluation of novel therapies (e.g., stem cell therapy) aimed at ameliorating the condition, given the high incidence of overt DCM and the insidious and more stable form of CHF that follows, which translates into increased survival at the end of the induction period. As a note added in proof, we have recently reported that, using the protocol of induction as in DAU4 group and successful induction of AICM, stem cell therapy with amniotic membrane-derived mesenchymal stem cells (AM-MSC), administered percutaneously via intramyocardial injection, significantly improved ventricular function at 2 and 4 weeks after transplant and also significantly improved survival compared with control group [43]. We also believe that the protocol used in DAU3 group is still very valuable in evaluation of novel cardioprotective drugs, aimed at preventing development AICM in the preclinical setting and serving as a model for the study of pathophysiological aspects of AICM.

## 5. Conclusion

Our results indicate that, compared to other protocols of induction of AICM (as in the DOX2 and DAU3 groups), a protocol using daunorubicin 4 mg/kg per week for 6 weeks (as in the DAU4 group) results in a high percentage of animals with overt DCM (92%) and CHF (61%), with mild manifestations of nonspecific systemic compromise, very low premature mortality (7.6%) during the induction period, and low cumulative mortality within the two weeks after completion of the induction protocol, which translated into a significantly higher survival rate at this stage, whilst development of CHF was more insidious (compared to the DAU3 group, in which abrupt development of CHF was quickly followed by death of the animals). We propose that this refinement of the model of AICM in rabbits, which translates into a more predictable cardiotoxicity, represents a useful tool for the preclinical evaluation of novel therapies (e.g., drugs or stem cell therapy) for the treatment of heart failure secondary to AICM, as well as the study of the molecular mechanisms involved in the development of AICM. This refinement is also in line with the guidelines of the National Centre for the Replacement, Refinement and Reduction of Animals in Research and the 3Rs principles, since the reduced nonspecific toxicity and lower mortality would ultimately translate into improved overall well-being of the animals and reduced amount of animals required for this type of research. However, we believe that the protocol used in DAU3 group is still very valuable in evaluation of novel cardioprotective drugs, aimed at preventing development AICM in the preclinical setting, and for the study of the pathophysiology of AICM.

## Supplementary Material

Supplemental Table 1. Selected biochemical parameters in Male and Female at final time point.
Supplemental Figure 1. Echocardiographic and cTnI values in Males and Females at final time point.

## Figures and Tables

**Table 1:** Selected biochemical parameters.

Parameter	Basal	Intermediate^§^	Final
Cholesterol (mg/dL)			
Control	41.5 ± 10.3	37.5 ± 10.2	44.7 ± 3.8
DOX2	36.10 ± 11.25	70.46 ± 16.66^bc#¶^	241.04 ± 59.5^bc#¶^
DAU3	40.80 ± 4.34	45.66 ± 6.40	79.73 ± 8.20^bc^
DAU4	35.24 ± 7.71	44.38 ± 4.05	59.08 ± 7.81^bc^
Triglycerides (mg/dL)			
Control	72.3 ± 22.9	45.3 ± 7.3	55.3 ± 7.1
DOX2	68.23 ± 17.48	184.70 ± 47.74^b¶^	405.88 ± 38.80^bc¶^
DAU3	78.09 ± 20.39	150.31 ± 65.32^b¶^	446.22 ± 102.9^bc¶^
DAU4	72.50 ± 22.85	85.04 ± 12.35	115.88 ± 15.72^bc^
Creatinine (mg/dL)			
Control	0.86 ± 0.10	0.77 ± 0.11	0.83 ± 0.10
DOX2	0.93 ± 0.06	1.08 ± 0.08	1.62 ± 0.22^bc^
DAU3	0.79 ± 0.04	1.06 ± 0.10	2.54 ± 0.50^bc*∗*¶^
DAU4	0.98 ± 0.10	0.92 ± 0.10	0.87 ± 0.07
BUN (mg/dL)			
Control	37.00 ± 4.95	38.54 ± 6.62	41.79 ± 8.50
DOX2	36.43 ± 3.45	30.50 ± 3.74	39.40 ± 8.69
DAU3	26.61 ± 2.99	30.92 ± 2.47	85.47 ± 12.34^bc*∗*¶^
DAU4	35.42 ± 4.57	28.35 ± 1.38	29.96 ± 2.50
Total Proteins (g/dL)			
Control	5.39 ± 0.21	5.37 ± 0.22	5.33 ± 0.24
DOX2	5.48 ± 0.17	4.90 ± 0.37	3.92 ± 0.37^bc¶^
DAU3	5.31 ± 0.17	5.90 ± 0.30	4.42 ± 0.81^bc^
DAU4	5.55 ± 0.16	5.20 ± 0.12	4.96 ± 0.17
AST (U/L)			
Control	23.02 ± 4.90	18.47 ± 4.10	20.77 ± 4.34
DOX2	27.59 ± 4.71	24.36 ± 6.86	30.42 ± 1.01
DAU3	20.96 ± 2.93	22.72 ± 5.82	270.9 ± 124.0^bc*∗*¶^
DAU4	35.94 ± 5.73	23.30 ± 3.84	28.31 ± 5.67
ALT (U/L)			
Control	44.67 ± 8.16	39.97 ± 7.89	37.45 ± 7.37
DOX2	53.02 ± 5.34	36.98 ± 7.45	35.46 ± 4.30
DAU3	48.75 ± 6.47	54.56 ± 12.34	226.70 ± 97.2^bc*∗*¶^
DAU4	58.25 ± 13.44	45.03 ± 5.96	42.19 ± 6.88

^§^Intermediate time point values were from blood samples obtained on week 8 of the study in control group, on week 6 in DOX2 group, on week 8 in DAU3 group, and on week 4 in DAU4 group. BUN, blood urea nitrogen; AST, aspartate aminotransferase; ALT, alanine aminotransferase. Data expressed as mean ± SEM. Statistical significance is at *p* < 0.05; ^b^compared to basal value; ^c^compared to control; ^*∗*^compared to DOX2; ^#^compared to DAU3; ^¶^compared to DAU4.

**Table 2:** Haematological parameters.

Parameter	Basal	Intermediate^§^	Final
RBC (10^6^/*μ*L)			
Control	5.95 ± 0.24	5.93 ± 0.12	6.12 ± 0.20
DOX2	6.06 ± 0.20	4.76 ± 0.26^bc^	5.44 ± 0.74
DAU3	6.29 ± 0.27	4.87 ± 0.19^bc^	3.83 ± 0.87^bc*∗*¶^
DAU4	6.33 ± 0.25	4.84 ± 0.20^bc^	5.23 ± 0.23
Haematocrit (%)			
Control	36.93 ± 1.76	36.88 ± 0.61	39.53 ± 0.90
DOX2	37.11 ± 1.75	28.42 ± 1.54^bc^	33.29 ± 2.67
DAU3	36.47 ± 1.10	29.56 ± 0.65	25.40 ± 3.56^bc*∗*¶^
DAU4	39.36 ± 0.98	29.16 ± 1.32^b^	33.94 ± 2.46
Hemoglobin (g/dL)			
Control	12.52 ± 0.47	11.75 ± 0.23	12.82 ± 0.30
DOX2	12.21 ± 0.63	9.14 ± 0.32^b^	10.40 ± 1.23
DAU3	12.18 ± 0.24	8.68 ± 0.24^b^	6.83 ± 1.08^bc*∗*¶^
DAU4	13.07 ± 0.44	9.19 ± 0.40^b^	9.57 ± 0.59^bc^
MCV (*μ*m^3^)			
Control	62.17 ± 2.12	62.35 ± 1.41	62.75 ± 1.78
DOX2	61.18 ± 1.87	59.95 ± 3.18	62.40 ± 3.27
DAU3	58.08 ± 1.52	60.88 ± 1.33	62.33 ± 5.03
DAU4	62.29 ± 1.75	60.28 ± 1.36	64.73 ± 3.15
RDW			
Control	13.37 ± 0.74	13.95 ± 0.72	13.42 ± 1.14
DOX2	13.08 ± 0.38	18.44 ± 1.15^bc^	17.32 ± 1.34^bc^
DAU3	13.48 ± 0.28	20.26 ± 0.83^bc¶^	20.47 ± 1.44^bc*∗*^
DAU4	12.27 ± 0.41	15.98 ± 0.76^bc^	19.78 ± 0.78^bc^
WBC (10^3^/*μ*L)			
Control	5.08 ± 1.22	4.86 ± 0.69	5.05 ± 0.82
DOX2	5.77 ± 0.99	6.52 ± 1.14	5.53 ± 0.76
DAU3	4.42 ± 0.64	2.88 ± 0.46^bc*∗*¶^	7.41 ± 0.62^bc*∗*^
DAU4	5.43 ± 0.63	4.80 ± 0.72^*∗*^	8.59 ± 1.92^bc*∗*^
Differential count (%)			
Neutrophils			
Control	15.55 ± 2.52	17.57 ± 1.91	22.78 ± 5.20
DOX2	18.20 ± 3.66	12.78 ± 2.49	25.70 ± 9.89
DAU3	14.10 ± 2.19	10.18 ± 4.35	11.10 ± 0.95
DAU4	31.16 ± 9.79	6.58 ± 1.60^bc^	27.44 ± 3.03
Lymphocytes			
Control	68.55 ± 5.25	65.48 ± 2.97	63.50 ± 4.68
DOX2	68.38 ± 4.55	70.63 ± 5.21	55.90 ± 10.34
DAU3	73.27 ± 2.67	76.52 ± 5.47	72.20 ± 1.30
DAU4	55.86 ± 8.73	82.56 ± 2.14	57.15 ± 3.47

^§^Intermediate time point values were from samples obtained on week 6 in DOX2 group, on week 8 in DAU3 group, and on week 4 in DAU4 group. RBC, red blood cells; MCV, mean corpuscular volume; RDW, red cell distribution width; WBC, white blood cells. Data expressed as mean ± SEM. Statistical significance is at *p* < 0.05; ^b^compared to basal value; ^c^compared to control; ^*∗*^compared to DOX2; ^¶^compared to DAU4.

**Table 3:** Scores for grade and extension of histopathological lesions in the heart.

Group	Lesion grade	Lesion extension
DOX2	2.65 ± 0.39	1.65 ± 0.44
DAU3	2.63 ± 0.43	2.68 ± 0.47^*∗*^
DAU4	2.76 ± 0.32	3.18 ± 0.43^*∗*#^

Data expressed as mean ± SEM. Statistical significance is at *p* < 0.05; ^*∗*^compared to DOX2; ^#^compared to DAU3.

**Table 4:** Scores for grade and extension of histopathological lesions in kidney and liver.

Group	Lesion grade	Lesion extension
Kidney		
DOX2	3.50 ± 0.71	3.50 ± 0.71
DAU3	2.75 ± 0.50	3.50 ± 0.58
DAU4	3.35 ± 0.57	3.17 ± 0.65

Liver		
DOX2	3.00 ± 0.10	3.00 ± 0.10
DAU3	3.00 ± 0.75	3.00 ± 0.71
DAU4	2.88 ± 0.90	3.04 ± 0.75

Data expressed as mean ± SEM.

## Abbreviations

AC: Anthracyclines
AICM: Anthracycline-induced cardiomyopathy
CHF: Congestive heart failure
DCM: Dilated cardiomyopathy
LVEF: Left ventricular ejection fraction
FS: Fractional shortening.
