# Supplementary material for: An Upgrade on the Rabbit Model of Anthracycline-Induced Cardiomyopathy: Shorter Protocol, Reduced Mortality, and Higher Incidence of Overt Dilated Cardiomyopathy
Source: Biomed Res Int. 2015 Dec 16;2015:465342. doi: 10.1155/2015/465342 (PMC4695679; doi:10.1155/2015/465342)
Supplement: Supplementary file 1 — Supplemental Table 1. Selected biochemical parameters in Male and Female at final time point. Supplemental Figure 1. Echocardiographic and cTnI values in Males and Females at final time point. [file 465342.f1.pdf]

Supplemental Table 1. Selected biochemical parameters in Male and Female at final time point.

Parameter	*Males*	*Females*
Cholesterol (mg/dL)		
Control	46.9 ± 13.1	41.5 ± 8.8
DOX2	252.70 ± 82.67 c¶	296.30 ± 64.03 c¶
DAU3	79.40 ± 11.10 n	80.05 ± 8.87 n
DAU4	55.07 ± 13.17 c	56.14 ± 15.83 c
Triglycerides (mg/dL)		
Control	65.9 ± 22.2	58.1 ± 12.3
DOX2	408.13 ± 49.09 c¶	388.82 ± 27.17 c¶
DAU3	436.66 ± 125.59 n	533.60 ± 66.77 n
DAU4	102.58 ± 26.04 c	118.99 ± 21.29 c
Creatinine (mg/dL)		
Control	0.84 ± 0.10	0.81 ± 0.06
DOX2	1.87 ± 0.33 c	1.46 ± 0.12 c
DAU3	2.66 ± 0.60 n	2.79 ± 0.53 n
DAU4	0.87 ± 0.19	1.05 ± 0.17
BUN (mg/dL)		
Control	36.99 ± 6.34	44.37 ± 8.75
DOX2	41.66 ± 8.21	38.58 ± 8.75
DAU3	79.60 ± 14.85 n	91.35 ± 20.59 n
DAU4	38.60 ± 5.40	36.85 ± 4.87
Total Proteins (g/dL)		
Control	5.22 ± 0.12	5.48 ± 0.22
DOX2	3.63 ± 0.19 c¶	3.78 ± 0.53 c¶

DAU3	4.66 ± 1.15 n	4.74 ± 1.31 n
DAU4	4.99 ± 0.50	4.88 ± 0.41
AST (U/L)		
Control	20.48 ± 4.23	26.33 ± 4.40
DOX2	29.78 ± 0.96	30.42 ± 1.02
DAU3	350.57 ± 187.7 n	356.40 ± 185.2 n
DAU4	26.62 ± 18.70	31.32 ± 14.85
ALT (U/L)		
Control	38.83 ± 8.83	51.12 ± 7.89
DOX2	33.46 ± 3.02	35.36 ± 4.40
DAU3	288.83 ± 116.2 n	289.57 ± 105.9 n
DAU4	49.27 ± 15.08	45.08 ± 3.39

BUN = blood urea nitrogen; AST = aspartate aminotransferase; ALT = alanine aminotransferase. Data expressed as mean ± SEM. Statistical significance: $p < 0.05$, (c) : compared to control; (*) : compared to DOX2; (¶) : compared to DAU4; (n) : low *n*, type II error.

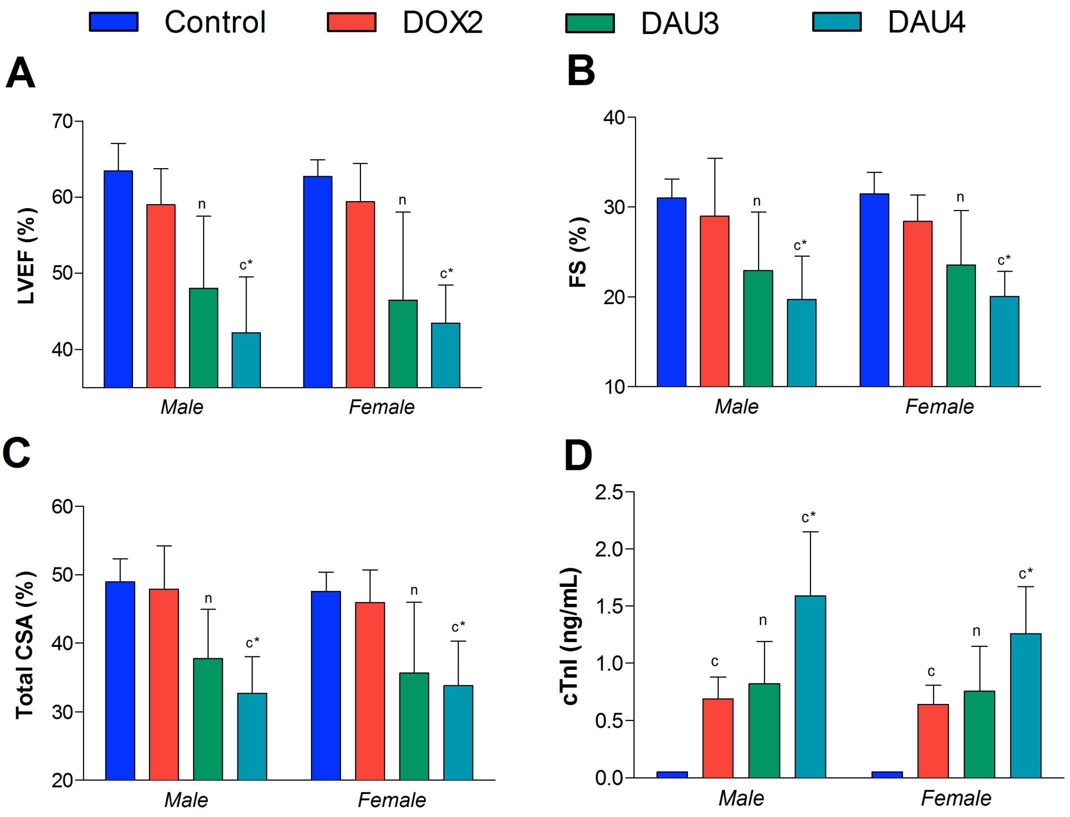

Supplemental Figure 1. Echocardiographic and cTnI values in Males and Females at final time point. (A). Left ventricular ejection fraction (LVEF); (B). Fractional shortening (FS); C. Total circumferential shortening area (CSA). (D). Cardiac Troponin I (cTnI) levels. Data expressed as mean ± SEM. Statistical significance: $p < 0.05$, (c) : compared to control; (*) : compared to DOX2, (n) : low n, type II error.
